# Repertoire of Therapeutics

**Published:** 1903-09

**Authors:** 


### Abstracts and Selections.

### Repertoire of Therapeutics.

Of all the agents advocated against tuberculosis, creosote is the only one which has been clinically and experimentally tested with success. What was taught to us by empirical medicine is strangely justified by the experiments of d'Arloing, who has demonstrated by sero-reaction that an organism submitted to the creosote treatment was manufacturing a defective toxin, able to influence in vitro the vitality of the bacillus of Koch.

But while it remains evident that creosote is the essential active principle against the bacillus of tuberculosis, the form under which it should be administered has always been discussed until now. Used in a crude state, creosote provokes gastric troubles which are attributed to the free phenolic functions making it caustic.

M. Ballard (Thesis, Montpellier, 1894), has succeeded in preparing the Phosphite of Creosote which he has called Phosphotal by making the trichloride of phosphorus act on sodic creosote. Thus is obtained a thick liquid, reddish-yellow, possessing a feeble odor of creosote, which contains 90 per cent of creosote and about 9 per cent of phosphorus, in the form of phosphorus acid.

The phosphorus is then absorbed in an assimilable state and rapidly regenerates the nervous tissue, while it transforms in resorbable fat the products of exudation of the pulmonary alveolæ.

Phosphotal, or ether of creosote, is not caustic, the proof of this is that even in heavy doses it does not produce gastro-intestinal disturbances. M. Ballard gave five grammes of phosphotal daily during three years to one of his patients who never was inconvenienced by it; this innoxiousness of the phosphite of creosote has been confirmed by the experiments of Vedel.

It will only be necessary to determine how long it takes for the elimination of phosphotal, so as to ascertain its absorption. A patient (Thesis Fouzes Diacon) takes an enema of 300 grammes of milk containing 1 gramme 20 of phosphotal, and although he retained it only one hour and a quarter, we find the creosote in the urine one hour and a half after the administration of the enema. On the following day more than one-half of the creosote given was found in the urine.

No preparation of creosote possesses such conclusive advantages.

Phosphotal may be prescribed in two different ways; either in utilizing the Capsules Clin au Phosphotal at the dose of from 4 to 12 capsules a day, or in compounding an enema with a teaspoonful of the Emulsion Clin au Phosphotal dissolved in a half glass of tepid milk.

The Capsules Clin are gluten coated: this substance, which is affected only in alkaline surrounding, goes through the stomach and is only transformed in the intestines by contact with the pancreatic juice. It results from this that the medicine administered to tuberculous patients in this shape can not deteriorate their stomach, which constitutes their only defense.—*Presse Medicale.*

